# A case report and literature review: leiomyosarcoma or perivascular epithelioid cell neoplasm?

**DOI:** 10.3389/fonc.2024.1499403

**Published:** 2024-12-20

**Authors:** Zheng Chen, Xuan Zheng, Qin Lin

**Affiliations:** ^1^ Department of Obstetrics and Gynecology, The International Peace Maternity and Child Health Hospital, School of Medicine, Shanghai Jiao Tong University, Shanghai, China; ^2^ Shanghai Key Laboratory of Embryo Original Diseases, The International Peace Maternity and Child Health Hospital, Shanghai, China; ^3^ Shanghai Municipal Key Clinical Specialty, The International Peace Maternity and Child Health Hospital, Shanghai, China

**Keywords:** case report, uterine neoplasm, leiomyosarcoma, perivascular epithelioid cell neoplasm, diagnosis

## Abstract

The distinction between a uterine leiomyosarcoma (uLMS) and a perivascular epithelioid cell neoplasm (PEComa) can be quite challenging. Here we report a 39-year-old woman who underwent a hysteroscopic myomectomy. An intraoperative frozen section pathological examination revealed that the mass was likely to be a mesenchymal malignancy. After consultation with her family, a total hysterectomy and bilateral salpingo-oophorectomy were performed. Postoperative pathological examinations suggested leiomyosarcoma but a malignant PEComa cannot be completely excluded. Combining the present case and prior studies, we summarized the clinical manifestations, pathological features, genomic characterization, and treatment of LMS and PEComa.

## Introduction

Leiomyosarcoma (LMS) is one of the most common malignant mesenchymal tumors and is composed of cells that exhibit distinct features of the smooth muscle lineage (1). LMS most commonly occurs in the uterus. A perivascular epithelioid cell neoplasm (PEComa) is a mesenchymal tumor that originates from histologically and immunohistochemically distinctive perivascular epithelioid cell (PEC) lines characterized by the coexpression of melanocytic and muscle markers (2). A PEComa arising from the female genital tract accounts for nearly 25% of all PEComa cases and the uterine corpus is the most commonly affected organ. The distinction between an LMS and a PEComa is quite challenging. In this study, we report the case of a 39-year-old woman and discuss the diagnosis and treatment of an LMS and a PEComa.

## Case report

**Figure 1:**
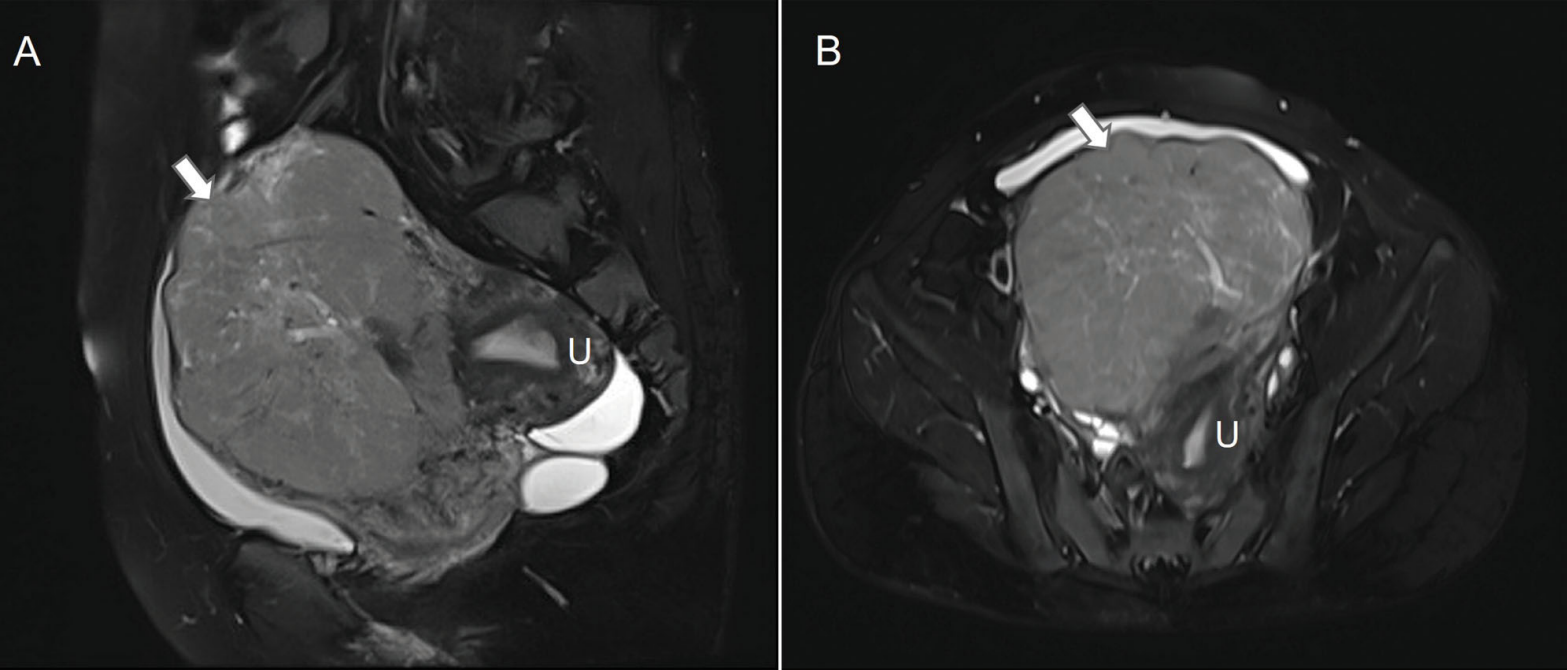
Pelvic magnetic resonance image of the mass in the uterus. On sagittal **(A)** and horizontal **(B)** T2-weighted images, the mass (arrows) abutted the uterus (U).

**Figure 2:**
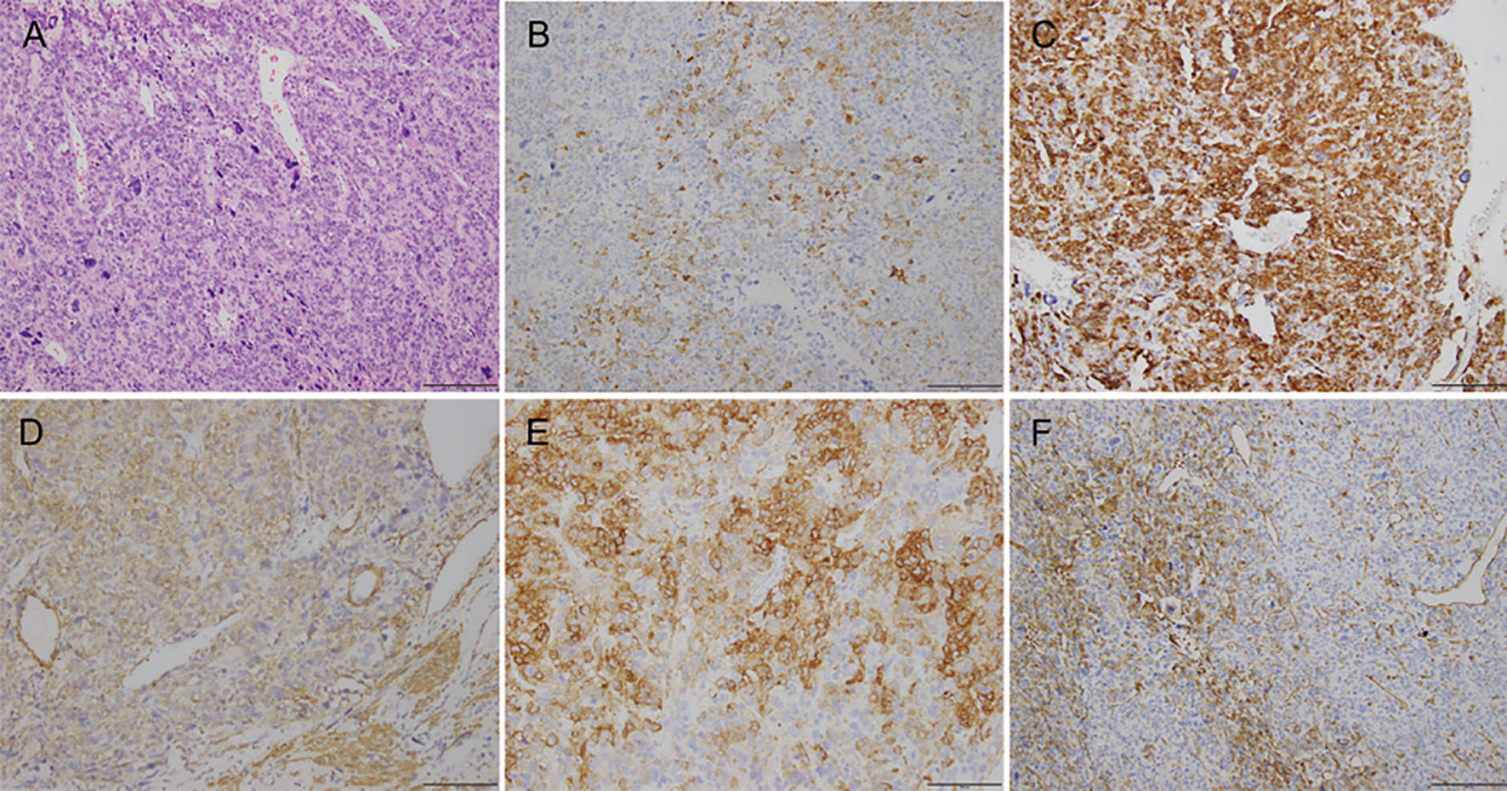
Photomicrographs showing **(A)** hematoxylin-eosin staining; **(B)** immunohistochemical staining for HMB45; **(C)** immunohistochemical staining for DES; **(D)** immunohistochemical staining for SMA; **(E)** immunohistochemical staining for H-caldesmon; **(F)** immunohistochemical staining for vimentin.

A 39-year-old woman (gravida 1, para 1) presented at our hospital with increased urinary frequency for the previous 3-4 months. The patient had no history of abnormal uterine bleeding, low abdominal pain, or weight loss. She had no significant medical, surgical, or family history. She had no history of smoking, drug use, or drinking. Serum levels of CEA, CA125, CA19-9, AFP, CA242, CA153, HE4, and NST were measured as part of a routine workup and the results were all negative. A gynecological examination revealed a uterine size as large as that observed at 18 gestational weeks. A gynecological B ultrasonic examination revealed an 11*10*9 cm low-echo mass arising from the anterior aspect of the uterus. Magnetic resonance imaging (MRI) revealed a 10*12*15 cm mass arising from the right anterior aspect of the uterus, and a subserosal uterine myoma with degeneration was diagnosed (**Figure 1**). On the basis of the preoperative examination results, a hysteroscopic myomectomy was performed first. A 12 cm mass was observed in the right anterior aspect of the uterus. The surface of the mass was covered with blood vessels. On gross examination, the mass was soft and fleshy. An intraoperative frozen section pathological examination revealed that the mass was likely to be a mesenchymal malignancy. After consultation with her family, a total hysterectomy and bilateral salpingo-oophorectomy were performed. Immunohistochemical staining revealed the expressions of HMB45 (partly+), SMA (+), DES (++), H-caldesmon (+), Vimentin (partly+), Ki67 (60%+), MyoD1 (cytoplasm+), INI-1 (+), BRG-1 (+), BRM (+), A103 (-), NL2 (focal+), EMA (partly+), caldesmon (++), ER (partly+), PR (partly+), CD10 (focal+), P53 (+), CD117 (-), CD31 (vessel+), CD34 (vessel+) and D240 (vessel+) (**Figure 2**). The histopathological diagnosis was suggestive of leiomyosarcoma, but a malignant PEComa could not be completely excluded. Genetic testing was recommended but unfortunately, the patient was lost to follow-up.

## Discussion

This case report shows interesting features that might help other gynecologists approach the diagnosis and treatment of an LMS or a PEComa.

Currently, no clinical symptoms can provide effective preoperative diagnostic modalities for an LMS or a PEcoma. Patients with an LMS present with abnormal vaginal bleeding, a palpable pelvic mass, and/or pelvic pain. Common symptoms of a PEComa include altered menstruation, irregular vaginal bleeding, abdominal pain, or the identification of a mass on imaging (2, 3). The diagnosis is not established until pathological and immunohistochemical analyses of the surgical specimen are carried out in a large percentage of cases. Our case only presented with a large pelvic mass with increased urinary frequency.

The preoperative diagnosis of a subserosal uterine myoma with degeneration was made by our radiologists via MRI. MRI has the greatest potential to be an effective screening tool for an LMS. Low ADC values, irregular margins, and T2 intermediate/hyperintensity are observed more often in patients with an LMS. The combination of low ADC values and the presence of an irregular margin may be a more specific characteristic, whereas the presence of a T2 intermediate/high signal may be more sensitive (4). Some reports have indicated that arteriovenous hypervascularity in contrast-enhanced computed tomography (CT) images may be a feature of a PEComa (5). Diagnosing LMS or a PEComa solely through preoperative imaging (including CT and MRI) is a challenging task.

Histopathological assessment is the standard method for the diagnosis of an LMS and a PEComa. The cut surfaces of an LMS are typically soft, bulging, fleshy, necrotic, hemorrhagic, and lack the prominent whorled appearance of a leiomyoma. LMS is classified into three subtypes: spindle-type, myxoid-type, and epithelioid-type (6). The cut surfaces of a PEComa are soft to firm and vary in color (pink, tan-brown, yellow-brown, white, gray-white), with a subset showing friability, hemorrhage, or necrosis. PEComa is generally classified into three subgroups: spindle-type, epithelioid-type, and admixed-type (7). The differential diagnosis is still challenging in terms of histopathology since LMSs and PEComas can both present with epithelioid, spindle-shaped or mixed pattern epithelioid and spindle-shaped cells. A delicate vascular network may be characteristic of a PEComa (8).

Because some of morphological features are not unique to an LMS compared with a PEComa, specific immunohistochemical stains are required to support the morphological diagnosis. The majority of LMSs are immunoreactive for α-smooth muscle actin (α-SMA), desmin, and h-caldesmon. Approximately one-third of leiomyosarcomas focally express HMB-45 (9). PEComas are immunoreactive for both smooth muscle (desmin, SMA, muscle-specific-actin, muscle myosin, and calponin) and melanocytic (HMB-45, Melan-A/MART-1, tyrosinase, and MITF) markers (10). The two main morphological subtypes of a gynecological PEComa have been described. One subtype, which is closely reminiscent of a low-grade endometrial stromal sarcoma, exhibits strong HMB-45 expression with only focal smooth-muscle marker positivity. The other type, which exhibits overlapping morphological and immunohistochemical features with epithelioid smooth muscle tumors, exhibits lower HMB-45 expression and more extensive smooth-muscle marker positivity. It has been proposed that two or more melanocytic markers (preferably HMB-45 and Melan A) or one melanocytic marker and cathepsin K are enough to indicate a diagnosis of PEComa (11). In the light of the immunohistochemical results of our patient—HMB45 (partly+), SMA (+), DES (++), and H-caldesmon (+)—the diagnosis remains uncertain.

Given the immunophenotypic overlap that exists between PEComas and LMSs, genomic characterization is needed for differential diagnosis. An LMS is a malignant mesenchymal neoplasm originating from smooth muscle cells. Exome sequencing has shown that TP53, RB1, ATRX, PTEN, and MED12 are frequently mutated in LMSs (12). A PEComa is a mesenchymal tumor thought to originate from PEC lines. Immunophenotypic and molecular overlap advocate for the hypothesis that modified smooth muscle cells represent the origin of a subset of PEComas (13). Recurrent alterations were also identified in TP53, RB1, ATRX, and BRCA2 in PEComas (14). TSC alterations/TFE3 fusions are characteristic of uterine PEComas (9). TFE3-rearranged PEComas were identified as a distinctive subset of PEComas. SFPQ was the most common TFE3 fusion partner, followed by the ASPSCR1 and NONO genes (15). A significant subset of PEComas show overexpression of MITF in the absence of TFE3 expression (16). Additionally, the novel RAD51B gene fusion is observed exclusively in uterine PEComas (17). Unfortunately, our case was not explored by further molecular analysis to help in the differential diagnosis.

For an LMS or a PEComa, the effective treatment is surgical removal with negative surgical margins including a total abdominal hysterectomy and bilateral salpingo-oophorectomy (10, 18). Although the risk of recurrence after complete resection of an LMS is high, adjuvant chemotherapy or radiation therapy administered after complete resection does not significantly improve the oncological outcome. For a PEComa, only anecdotal and inconclusive data are available for the use of adjuvant radiotherapy.

## Conclusion

In summary, we present an unusual case that cautions that large pelvic masses may present atypically. Uterine PEComa cases are rare. One type of uterine PEComa has morphological and immunohistochemical features that overlap those of an LMS and should be considered in the differential diagnosis. We hope that this case may contribute to increasing the awareness of PEComas and ultimately to avoiding clinical misdiagnosis, missed diagnosis, and treatment delay.

## Data Availability

The raw data supporting the conclusions of this article will be made available by the authors, without undue reservation.
